# AN INTELLIGIBLE AI-DRIVEN DECISION SUPPORT SYSTEM FOR POSTSTROKE MOBILITY ASSESSMENT

**DOI:** 10.2340/jrm-cc.v8.42379

**Published:** 2025-07-20

**Authors:** Jin Cheng LIAW, Dominik RAAB, Malte WEBER, Mario SIEBLER, Harald HEFTER, Dörte ZIETZ, Marcus JÄGER, Andrés KECSKEMÉTHY, Francisco GEU FLORES

**Affiliations:** 1 Chair of Mechanics and Robotics, University of Duisburg-Essen, Duisburg, Germany; 2 Mediclin Rehabilitationsforschung gGmbH, Offenburg, Germany; 3 Department of Neurology, University Hospital Düsseldorf, Düsseldorf, Germany; 4 Department of Applied Health Sciences – Physiotherapy, University of Applied Sciences Bochum, Bochum, Germany; 5 Department of Orthopedics, Trauma and Reconstructive Surgery, St. Marien-Hospital Mülheim an der Ruhr, Mülheim an der Ruhr, Germany; 6 Chair of Orthopaedics and Trauma Surgery, University of Duisburg-Essen, Germany

**Keywords:** automated poststroke mobility assessment, decision trees, deep learning, gait analysis, stroke rehabilitation

## Abstract

**Objective:**

Long-term mobility impairment is a sequel of stroke victims which requires intensive medical and physiotherapeutic care. Detailed assessment of therapeutic success is relevant to achieving efficacy, but requires expert knowledge, since mobility disorders are complex. Increasing shortage of qualified staff and larger numbers of patients are thus major problems in this field. To meet these challenges, we show that machine learning algorithms can reproduce expert mobility assessment from gait data with acceptable accuracy, supporting poststroke evaluation while giving intelligible feedback into how the assessments were generated.

**Methods:**

A total of 100 hemiparetic stroke patients received clinical examinations followed by instrumented gait analysis and were assigned a Stroke Mobility Score by an interdisciplinary expert board. From each measured stride pair, 680 features were extracted. After removing non-discriminating features, two regression models were trained: a decision tree and a multilayer perceptron artificial neural network.

**Results:**

The models yielded good to very good (Cohen) coefficients of determination. The interpretable decision-trees and the explanations obtained from the neural network unveiled key features supporting the mobility assessments.

**Conclusion:**

The automated assessments agree well with those of the experts. Synergistic interactions between system, and experts via the computed key features may improve quality in diagnosis and objectify therapeutic targets.

## LAY ABSTRACT

One major impairment caused by stroke is an affected walking ability, which is strongly dependent on the location and extent of the damage. Because of the wide spectrum of complex pathological movement patterns, patients benefit from personalized rehabilitation programs. Mobility assessment by experts from different medical disciplines has been shown to contribute considerably to these objectives. We present machine-learning models that can reconstruct expert mobility assessment. Moreover, we demonstrate that the “reasoning” of the models can be explained to an important extent. This allows the models to communicate with the experts and hence support them by granting them quantitative access to their own qualitative reasoning. This synergy could improve the long-term performance of both models and experts, thus leading to a more objective and effective rehabilitation therapy.

Mobility recovery and, hence, accurate mobility assessment, is essential in poststroke rehabilitation (1, 2). Currently, poststroke mobility is assessed via medical history and qualitative observational examinations performed by trained clinicians from different disciplines (neurology, orthopaedics, physiotherapy, orthotics). Such subjective examinations may yield weak reliability, sensitivity, and specificity (3), since they are based on the discipline-specific expertise of each clinician (4). Moreover, such interdisciplinary assessments are too resource-intensive to be applicable for therapeutic monitoring.

Instrumented gait analysis allows for an objective and reliable mobility examination via spatiotemporal, kinematic, and kinetic gait measurements (3). While accurate motion-capture systems currently require a laboratory and trained personnel, current trends promise easy-to-use wearables that yield similar data from simpler measurements (3). However, the translation of gait data into medical assessments remains a challenge because of the data’s high resolution and dimensionality, which traditional statistical approaches are unable to handle (5). Artificial intelligence (AI) systems are a promising tool for meeting this challenge (3, 6–8).

In medical applications, however, there is a sensible reluctance to use AI systems for diagnosis due to their lack of interpretability (9–11). Explainable artificial intelligence techniques are emerging as an interface between AI systems and medical experts. Some examples are described in (10, 12, 13). These systems have the potential not only to support the user but also to offer an objective insight into the user’s own reasoning, which might enhance the user’s own assessments. In fact, AI may present to be an indispensable training tool, as it does for non-medical purposes such as gaming (14).

This study tests the performance and interpretability/explainability [as defined in (13)] of AI-driven poststroke mobility assessment based on instrumented gait analysis and interdisciplinary expert knowledge. To this end, the gait of 100 hemiparetic stroke patients was measured using a motion-capture system and video cameras, simultaneously. The video material was evaluated by a medical board of interdisciplinary experts via the Stroke Mobility Score (SMS), a multiple-cue observational clinical score designed for this purpose (2). The motion-capture data was used to train an interpretable decision-tree (DT) regression model as well as an explainable multilayer perceptron (MLP) artificial neural network to reproduce the expert board assessments from the measurements.

## METHODS

### Participants and gait data collection

A total of 100 hemiparetic stroke patients underwent a standard neurological examination and a full-body instrumented gait analysis, carried out on a straight 10-meter indoor walkway with a Vicon 3D motion capture system consisting of 10 marker-tracking cameras (100 Hz, Oxford Metrics Ltd., Oxford, England), and two video cameras (100 Hz, Basler AG, Ahrensburg, Germany) aligned along and perpendicular to the walkway. Reflective markers were attached to the patient according to the full-body Plug-in-Gait marker set (15). During the recordings, the patients walked at a self-selected pace barefoot or with shoes, wearing only underwear, and if necessary, aided with a walking cane or an ankle-foot orthosis (AFO). For each patient, at least four trials were recorded, each trial containing at least two consecutive strides.

The data was collected within 2 multidisciplinary research projects: “ReHabX-Stroke: Personalized therapy planning of gait disorders based on the example of stroke” (2012–2015) (16) and “RehaBoard: A computer assistance system for the interdisciplinary treatment planning of gait impairments after stroke” (2017–2020) (17). The inclusion criteria for the study population can be found in (2), where the same database is used to develop the SMS.

### Gait data processing

The motion-capture data was processed by the software Vicon Nexus 2 and post-processed by the software MobileBody (18). Nexus 2 delivered all Plug-in-Gait segment poses as well as the standard gait events (foot strike, foot off) while MobileBody interpolated the absolute pose of the Plug-in-Gait segments and computed the relative angles at the biomechanical joints, as well as their derivatives with respect to the gait-cycle progress. These derivatives correspond to the normalized angular velocities (NAVs) first introduced in (19). A NAV quantifies the slope of the tangent to the corresponding relative-angle progression, thus describing the shape of its plot.

### Expert board mobility assessment

The video camera data was evaluated by a medical board consisting of 5 experts from the fields of neurology (2x), orthopedics, physiotherapy, and orthotics. The expert board assigned each patient an SMS. As described in (2), the SMS is composed of six subscores corresponding to the functional criteria [1] trunk posture, [2] leg movement, [3] arm movement, [4] gait speed, [5] gait fluency, and [6] stability of walking on flat ground /risk of falling, which are here referred to as the Trunk-, Leg-, Arm-, Speed-, Fluency-, and Stability-SMS. Each subscore comprises 4 simple scoring descriptions from 0 (no pathological findings) to 3 (significant pathological findings), yielding an SMS lying between 0 (no findings) and 18 (most critical). After inspecting the video recordings (frontal- and sagittal-plane views) of a patient’s trial selected by chance, each expert board member recommended a value for every subscore. The expert board subscore was computed as the mode of all expert recommendations. If the mode could not be defined, the subscore not in contention for the highest count was used as a tiebreaker. The tiebreaker either served as a compromise, if situated between the other subscores sharing the highest count, or as a weight, if found on one side of the balance. For example, the subscores {1, 1, 2, 3, 3} yield an expert board subscore of 2, the subscores {0, 1, 1, 2, 2} yield a 1, and the subscores {1, 1, 2, 2, 3} yield a 2.

### Reconstruction of the expert board mobility assessment

Even though the expert board mobility assessment was performed at patient level by considering only one trial per patient, the machine-learning (ML) models were trained at stride pair level to use as many datapoints as possible. Every measured pair of consecutive ipsilateral and contralateral strides (stride pair) was coupled with the corresponding patient’s expert board subscores. The ipsilateral and contralateral sides of each patient were obtained from the case report files. The reconstruction of each subscore was performed as follows.

**Fig. 1:**
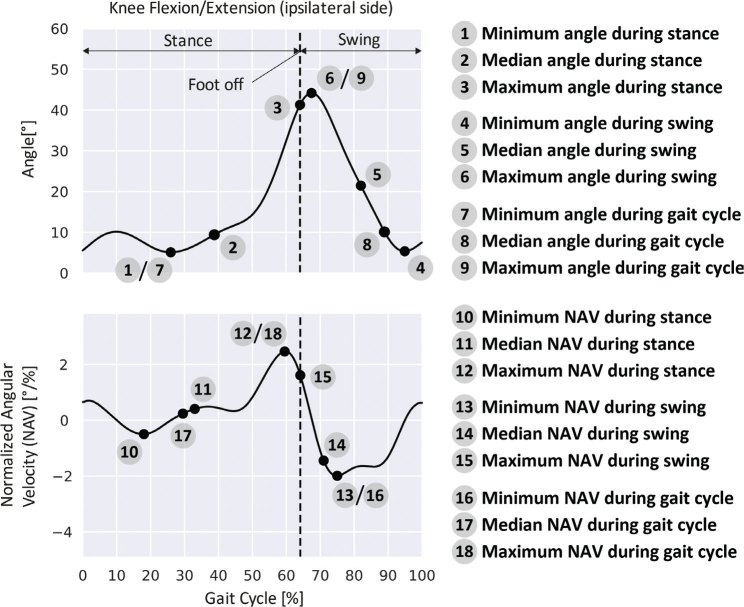
Example of the numerical characterization (19) of the knee flexion/extension normalized with respect to a patient’s ipsilateral stride

*Feature extraction.* A total of 339 features were extracted from the gait data of each stride. They comprise 15 gait parameters (12 standard gait parameters according to (20, 21) and in addition the duration of the stance phase as well as start and duration of the swing phase) and 324 features describing the relative angles at the biomechanical joints. The latter correspond to the numerical characterizations of movement introduced in (19) and are based on the descriptive statistics (minimum, median, and maximum values) of the relative angles and their corresponding normalized velocities, at the stance phase, swing phase, and the whole gait cycle (Fig. 1). Additionally, the usage of a walking aid was characterized with a 0 if no walking aid was used, and otherwise with a 1. The resulting 680 features for each stride pair are summarized in Table I.

**Table I:** Feature-extraction overview and manual feature selection (step 1) for each SMS subscore

*Extracted features*					*Feature selection (step 1)*					
*680 features per stride pair:• 30 gait parameters (15 parameters × 2 sides)• 648 numerical characterizations of movement (18 angles x 18 numerical characterizations × 2 sides)• 2 walking-aid features*					*Trunk-SMS*	*Leg- SMS*	*Arm-SMS*	*Speed-SMS*	*Fluency-SMS*	*Stability-SMS*
***Gait parameters***	*Standard*	Gait speed		[m/s]	X	X	X	X	X	X
		Cadence		[steps/s]	X	X	X	X	X	X
		Stride and step time		[s]	X	X		X	X	X
		Single and double support duration time		[s]	X	X		X	X	X
		Single and double support duration		[%]	X	X		X	X	X
		(Height) Normalized step width		[-]	X	X		X	X	X
		(Leg length) Normalized step and stride lengths		[-]	X	X		X	X	X
		Limp index		[-]	X	X		X	X	X
	*Phase*	Start of swing phase		[%]	X	X		X	X	X
		Duration of stance and swing phases		[%]	X	X		X	X	X
***Numerical characterization of movement***		Pelvis Orientation	Tilt, Obliquity, Rotation		X	X			X	X
		Thorax Orientation	Tilt, Side Tilt, Rotation		X		X		X	X
		Foot Orientation	Progression		X	X			X	X
		Hip Joint	Flexion/Extension, Adduction/Abduction		X	X			X	X
		Knee Joint	Flexion/Extension		X	X			X	X
		Ankle Joint	Dorsiflexion/Plantarflexion, Inversion/Eversion		X	X			X	X
		Spine	Tilt, Side Tilt, Rotation		X		X		X	X
		Shoulder Joint	Flexion/Extension, Adduction/Abduction		X		X		X	X
		Elbow Joint	Flexion/Extension		X		X		X	X
***Aid***		Walking Cane	[-] {0: no usage, 1: usage}		X	X	X	X	X	X
		AFO	[-] {0: no usage, 1: usage}		X	X	X	X	X	X

SMS: Stroke Mobility Score; AFO: Ankle-foot orthosis.

*Training and test datasets.* The total dataset was split into training (70%) and test (30%) datasets in a stratified manner to reduce class imbalances (22), using the SMS to define the strata while ensuring that all the stride pairs of each patient are contained in only one of the datasets.

*Feature selection.* Feature selection was performed in two steps. In a first step, expert knowledge was used to trim the feature set for each of the subscores based on their functional definitions (2), as shown in Table I. In a second step, the reduced feature sets were filtered using a measure of the strength with which each feature characterizes the subscore values assigned by the expert board. To this end, a representative stride pair was selected for every patient in the training dataset according to (23) to avoid selection bias (24). The selected stride pairs were grouped according to the subscore value assigned by the expert board to the corresponding patient, and the resulting groups were tested for statistical differences between the group mean values using an Alexander-Govern (AG) test (25). Features with p-values lower than 5% were retained in the reduced feature set.

*Model selection.* The model hyperparameters were selected by evaluating each hyperparameter combination (grid search) with a 10-fold cross-validation to select the hyperparameters that maximize the cross-validation estimate of the coefficient of determination *R*^2^. At each cross-validation iteration, the representative stride pairs used during feature selection were excluded from the held-out validation datasets and instead reinserted into the training dataset to obtain an unbiased estimate while minimizing data loss (24). Furthermore, each stride pair was weighted to account for two sources of biases: the imbalanced distribution of the subscores, and the inhomogeneous number of stride pairs from each patient. To this end, the weight of each stride pair was computed as the ratio of the greatest number of stride pairs with the same subscore to the number of stride pairs with the stride pair’s subscore, divided by the number of stride pairs from the corresponding patient.

The DT model was implemented with scikit-learn (26) and is based on Breiman’s Classification and Regression Trees (27). The MLP model was implemented using the Tensorflow (28) framework, and Keras library via scikeras (26). The details of the procedure used for hyperparameter tuning are described in Table II.

**Table II:** Grid-search hyperparameter tuning of the decision-tree and multilayer perceptron regression models via a 10-fold cross validation procedure for each combination of hyperparameters. ReLU was used as activation function for every hidden unit of the multilayer perceptron model

RM	Model hyperparameter	Abbreviation	Values examined
*DT*	Number of features to consider during splitting at each tree node	DT-HP1	{*n*, $\sqrt{n}$, log *n*}
	Strategy used to choose the split at each tree node	DT-HP2	Select split that best minimizes mean squared error from {all possible splits, randomly initialized splits}
	Minimum weight fraction at each tree node	DT-HP3	{0.00, 0.01, 0.02, … , 0.05}
	Maximum depth of the trees	DT-HP4	{3, 4, 5} as recommended by (13)
MLP	Number of hidden layers	MLP-HP1	{2, 3} with hidden units {16, 8} and {32, 16, 8}, respectively
	Activation function of the last output unit	MLP-HP2	{linear, ReLU}
	Learning rate of the Adam optimizer (29)	MLP-HP3	{1 × 10^−3^, 1 × 10^−4^, 1 × 10^−5^} If 1 × 10^−5^, try the following values for MLP-HP1 and MLP-HP2 fixed: {7.5 × 10^−5^, 5.0 × 10^−5^, 2.5 × 10^−5^, 1.0 × 10^−5^, 7.5 × 10^−6^, 5.0 × 10^−6^, 2.5 × 10^−6^}

DT: Decision tree; MLP: Multilayer perceptron artificial neural network; RM: Regression model; *n*: Total number of features; ReLU: Rectified linear unit.

*Model training and testing.* The optimal DT and MLP models were trained with the training dataset, then tested on the test dataset at patient level, since the expert board subscores are meant to assess patients, not single stride pairs. To this end, the model outputs (subscores) were averaged across each patient’s stride pairs. If the subscore predictions lied outside their definition range, they were cut to the nearest boundary. The SMS predictions were computed as the sum of all the corresponding range-adjusted subscore predictions. Model performance was evaluated by comparing the expert board assessments with predictions, using the coefficient of determination *R*^2^ as performance metric.

*Model interpretation and explanation.* The optimal DT models were visualized as tree-structured decision chains allowing for interpretation, whereas the optimal MLP models were explained using permutation importance, which estimates feature importance by randomly shuffling the values of a feature and observing the resulting degradation of the model’s performance (26).

## RESULTS

A total of 100 patients were included in this study, from which a total of 904 stride pairs were extracted. The training and test dataset consist of 633 stride pairs of 65 patients and 271 stride pairs of 35 patients, respectively. All the steps were carried out on a 5.3 GHz Intel^®^ Core™ i9-10900K with Python 3 and other libraries for applications in science and data analysis (e.g. SciPy, NumPy, pandas) (26, 28, 30–32).

Based on objective data from gait analysis and input from an interdisciplinary expert board, we trained ML models that predict SMS subscores with a large correlation (33) to the expert board assessments (*R*^2^ > 0.25). The optimal model hyperparameters and the performance estimates are shown in Table III.

**Table III:** Optimal model hyperparameters of each SMS-subscore decision-tree and multilayer perceptron regression models, their performance on the test dataset in terms of the coefficient of determination *R*^2^, and the corresponding interrater reliabilities ICC_1.1_ of the expert board assessments

SMS subscore	Feature subset	Optimal Model Hyperparameters		*R* ^2^		ICC_1.1_
		DT	MLP	DT	MLP	Board
Trunk posture	241 of 680 features	DT-HP1: *n*	MLP-HP1: 3	0.37	0.58	0.65
		DT-HP2: Random	MLP-HP2: linear			
		DT-HP3: 0.03	MLP-HP3: 2.5e-05			
		DT-HP4: 5				
Leg movement	188 of 356 pre-selected features	DT-HP1: *n*	MLP-HP1: 3	0.51	0.59	0.73
		DT-HP2: Random	MLP-HP2: linear			
		DT-HP3: 0.02	MLP-HP3: 1e-05			
		DT-HP4: 5				
Arm movement	99 of 330 pre-selected features	DT-HP1: *n*	MLP-HP1: 3	0.40	0.61	0.72
		DT-HP2: Random	MLP-HP2: ReLU			
		DT-HP3: 0.03	MLP-HP3: 1e-03			
		DT-HP4: 5				
Gait speed	31 of 32 pre-selected features	DT-HP1: *n*	MLP-HP1: 2	0.77	0.77	0.72
		DT-HP2: Random	MLP-HP2: linear			
		DT-HP3: 0.03	MLP-HP3: 1e-04			
		DT-HP4: 5				
Gait fluency	263 of 680 features	DT-HP1: *n*	MLP-HP1: 2	0.54	0.76	0.72
		DT-HP2: Random	MLP-HP2: linear			
		DT-HP3: 0.01	MLP-HP3: 1e-03			
		DT-HP4: 5				
Stability of walking on flat ground/risk of falling	238 of 680 features	DT-HP1: *n*	MLP-HP1: 2	0.86	0.85	0.83
		DT-HP2: Random	MLP-HP2: linear			
		DT-HP3: 0.02	MLP-HP3: 1e-03			
		DT-HP4: 5				
Collection of SMS subscore models combined to predict the SMS				0.82	0.87	0.88

SMS: Stroke Mobility Score; DT: Decision tree; MLP: Multilayer perceptron artificial neural network; *R*^2^: Coefficient of determination; DT-HP1, DT-HP2, DT-HP3, DT-HP4, MLP-HP1, MLP-HP2, MLP-HP3: See Table II.

**Fig. 2:**
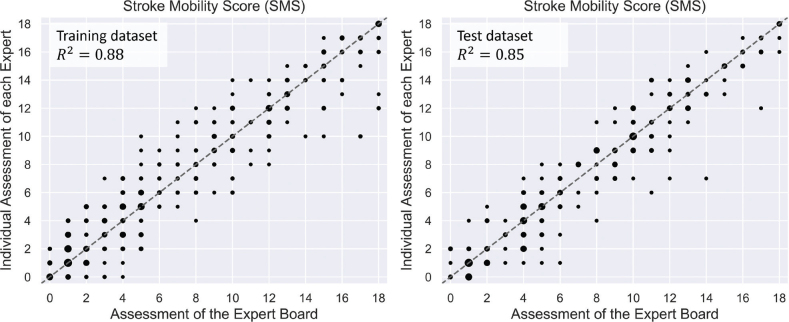
Scatterplots showing how the individual experts compare with the expert board mobility assessment (abscissa) for the SMS of the training dataset (left) and the test dataset (right)

**Fig. 3:**
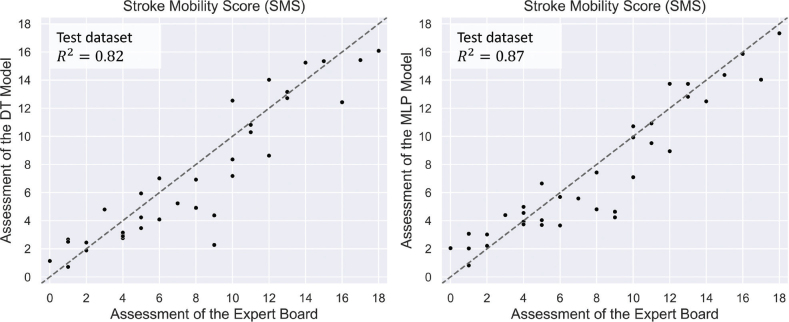
Scatterplots showing how the trained decision-tree models (left) and multilayer perceptron models (right) compare with the expert board mobility assessment (abscissa) for the Stroke Mobility Score

The overall performance of the models is comparable to the agreement of each expert recommendation with the collective decisions of the expert board (Figs 2 and 3).

**Fig. 4:**
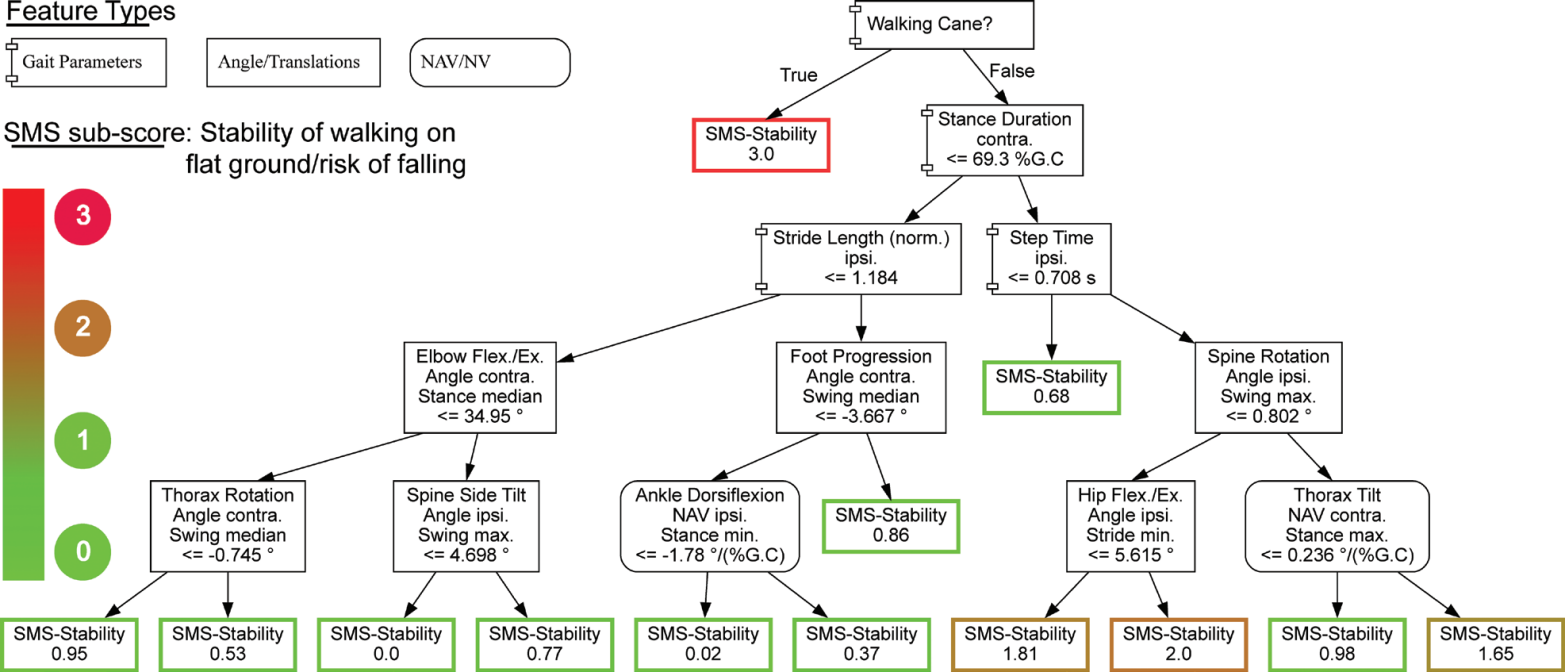
Decision tree model predicting for the Stroke Mobility Score –Stability

The DT models allow for a straightforward interpretation of the model decision-making process. Figure 4 presents the DT model for the Stability-SMS as an example. The DT models predicting all the SMS subscores are shown in Appendix A. The key features of the DT and the MLP models for the Stability-SMS are shown in Table IV. The key features of all SMS subscores can be found in Appendix A.

**Table IV:** Top key features used by the MLP and decision-tree regression models for the SMS-Stability, sorted from most to least important. The features used by the multilayer perceptron models were ranked according to permutation importance with 500 repetitions of random shuffling. The features used by the decision-tree models were sorted from most to least important in terms of their total contribution in reducing the squared error over all tree splits

SMS subscore	Top 20 features used by the MLP model	All features used by the DT model (MLP rank in parenthesis)
Stability of walking on flat ground/risk of falling	1. Walking Cane?	1. (1) Walking Cane?
	2. Knee Flex./Ex. NAV ipsi.(Stance max.)	2. (221) Stance Duration contra.
	3. Hip Flex./Ex. NAV contra.(Stance max.)	3. (91) Step Time ipsi.
	4. Knee Flex./Ex. NAV contra.(Swing median)	4. (201) Spine Rotation Angle ipsi.(Swing max.)
	5. Ankle Dorsiflexion NAV ipsi.(Stance min.)	5. (125) Stride Length (norm.) ipsi.
	6. Ankle Dorsiflexion NAV ipsi.(Stride min.)	6. (79) Spine Side Tilt Angle ipsi.(Swing max.)
	7. Elbow Flex./Ex. NAV contra.(Swing median)	7. (121) Thorax Tilt NAV contra.(Stance max.)
	8. Knee Flex./Ex. NAV ipsi.(Stride max.)	8. (26) Foot Progression Angle contra. (Swing median)
	9. Elbow Flex./Ex. NAV contra.(Swing min.)	9. (228) Elbow Flex./Ex. Angle contra. (Stance median)
	10. Knee Flex./Ex. NAV ipsi.(Swing max.)	10. (136) Thorax Rotation Angle contra. (Swing median)
	11. Hip Flex./Ex. NAV contra.(Stride max.)	11. (5) Ankle Dorsiflexion NAV ipsi.(Stance min.)
	12. Shoulder Flex./Ex. NAV ipsi.(Swing max.)	12. (58) Hip Flex./Ex. Angle ipsi.(Stride min.)
	13. Hip Flex./Ex. NAV contra.(Swing max.)	-
	14. Foot Progression NAV ipsi.(Swing median)	-
	15. Hip Flex./Ex. NAV ipsi.(Swing max.)	-
	16. Knee Flex./Ex. NAV ipsi.(Stride min.)	-
	17. Shoulder Flex./Ex. NAV contra.(Stance median)	-
	18. Knee Flex./Ex. NAV ipsi.(Stride median)	-
	19. Elbow Flex./Ex. NAV contra.(Stride max.)	-
	20. Pelvis Rotation NAV contra.(Stance median)	-

SMS: Stroke Mobility Score; DT: Decision tree; MLP: Multilayer perceptron artificial neural network; NAV: Normalized angular velocity.

The expert board assessments and the predictions of the trained models show a high degree of conformity. The key features delivered by the models hint at where to look in detail when assessing the mobility of a poststroke patient. For example, when assessing the risk of falling, the stance duration as well as the maximal rate of change (NAV) of the ipsilateral knee flexion and the contralateral hip flexion angles during stance are most relevant (Table IV).

## DISCUSSION

The SMS and the Stability-SMS models perform very well (*R*^2^ > 0.8), while the other models perform well (*R*^2^ > 0.5) (Table III). The performances correlate strongly [Cohen (33), *p* = 0.02, *r* = 0.83] with the corresponding inter-rater reliabilities ICC_1.1_. A high ICC_1.1_ implies a high degree of agreement among the experts, thus naturally resulting in models with higher robustness and consistency with ground truth.

Not all individual subscore models performed perfectly well. However, they compensated each other for the SMS. Using similar feature extraction and selection methods, we experimented with DT and MLP models that directly predict the SMS. While the DT model performed slightly worse (*R*^2^ = 0.79), the MLP model showed a similar performance (*R*^2^ = 0.87).

The DT models produced two underpredicted outliers (see Fig. 3 at an expert board mobility assessment of 9). As explained in detail in Appendix B, both patients exhibit outlying walking speeds, which led to a false estimation of the Leg-SMS and Arm-SMS scores.

The overall key features consist of 46% ipsilateral and 54% contralateral features for the DT models, and 53% ipsilateral and 47% contralateral features for the MLP models. This shows the importance of regarding the contralateral side, which is often neglected during patient assessment and stroke rehabilitation (34, 35), thus confirming studies that show bilateral impairment of the upper and lower extremities poststroke (36). Furthermore, the NAVs make up a large percentage of the top features, namely 20 and 73% for the DT and MLP model, respectively. This suggests that not only descriptive values but also the shape of the joint angle progressions are important for mobility assessment.

In this work, key features were unveiled globally, that is, for the whole training dataset. It is also possible to use local explainers to obtain key features with respect to a particular stride pair (13, 37, 38). An example on how Shapley Additive Explanations (SHAP) may be used can be found in Appendix C. Together, global and local key features mirroring expert assessment can improve the understanding of mobility disorder.

In conclusion, the SMS prescribed by an interdisciplinary expert board can be reproduced very well from gait data using interpretable DT and explainable MLP models.

The SMS has proven to be an excellent score for AI-driven poststroke mobility assessment based on gait data. Its subscore structure allows for a nuanced clinical assessment of the different functional aspects of gait, which supports a detailed interpretation/explanation of the AI models.

Overall, this work shows that it is possible to build an intelligible AI-driven decision support system for poststroke mobility assessment. We believe that intelligibility is the key to a successful deployment of such a system, since the identification of the key features used by the AI models to make their predictions may lead to a more objective and effective identification of therapeutic targets.
